# About the Formation of NH_2_OH^+^ from Gas Phase Reactions under Astrochemical Conditions

**DOI:** 10.3390/molecules28072932

**Published:** 2023-03-24

**Authors:** Gabriele Dilena, Simone Pistillo, Enrico Bodo

**Affiliations:** Chemistry Department, University of Rome “La Sapienza”, 00185 Rome, Italy

**Keywords:** interstellar medium, gas-phase chemistry, hydroxylamine

## Abstract

We present here an analysis of several possible reactive pathways toward the formation of hydroxylamine under astrochemical conditions. The analysis is based on ab initio quantum chemistry calculations. Twenty-one bimolecular ion–molecule reactions have been studied and their thermodynamics presented. Only one of these reactions is a viable direct route to hydroxylamine. We conclude that the contribution of gas-phase chemistry to hydroxylamine formation is probably negligible when compared to its formation via surface grain chemistry. However, we have found several plausible gas-phase reactions whose outcome is the hydroxylamine cation.

## 1. Introduction

Hydroxylamine (NH_2_OH) is considered as a key intermediate in the prebiotic synthesis of ribonucleotides [1,2,3]. Despite being a long sought after molecule by astronomers [4], its observation [5] in the interstellar medium (ISM) is only very recent. The NH_2_OH molecule has been observed only once in the G+0.693-0.027 molecular cloud, located in the Sgr B2 region near the center of our galaxy. The latter is one of the chemically richest regions ever observed, and a continuous source of new (and also rather complex [6]) molecular species. In particular, G+0.693-0.027 appears to possess a very rich nitrogen chemistry with the synthesis of several pre-biotic chemical species such as urea [7]. The discovery of such molecules concentrated in a specific portion of the interstellar medium support hypotheses such as “RNA-world”, where the pre-biotic ingredients are supposed to be formed in space [8,9,10,11], especially those containing the biologically all-important N–O bond [12].

The formation of saturated molecular species in the ISM is often assumed to take place on the surfaces of icy grains that are a natural reservoir of weakened, reactive molecular hydrogen [13]. The route to the formation of NH_2_OH catalyzed by interstellar ices in molecular clouds conditions has been demonstrated recently [14,15,16]. However, in order to be detectable, the newly formed molecule must migrate from the grain to the gas phase. This is usually assumed to be driven by thermal desorption [13]. It has been shown that adsorbed NH_2_OH, in the presence of water, dissociates back to ammonia and hydrogen peroxide [4]. It therefore follows that, although the present evidence points to grain-surface chemistry as the main route to hydroxylamine formation, it might be of interest to assess whether other reactive channels are available to produce it directly in the gas phase.

The most obvious sources of hydroxylamine in the gas phase could be thought to be direct condensation reactions between oxygen and ammonia (O + NH_3_) [17], and between hydroxyl and amino radicals (OH + NH_2_) [18]. Unfortunately, both these reactions are unsuitable, because due to their stoichiometry, in order to stabilize the condensation product, they would require the participation of a third body that removes the excess energy. Three-body collisions, however, are unlikely events in a low-density environment, even in dark molecular clouds. In other words, in both these reactions, hydroxylamine appears as an intermediate complex, but not as the final product. Moreover, in order to proceed directly to the final closed shell species, the reaction between O + NH_3_ requires either passage through an intersystem crossing, or an excited state of the oxygen atom (^1^Δ, ~1 eV above the triplet ground state) that cannot be easily produced at low temperatures. The reaction involving the ammino radical instead proceeds either on a singlet or on a triplet surface, but both seem to produce only very stable by-products such as water, ammonia, or molecular hydrogen [18]. To the best of our knowledge, no further attempts to describe the formation of NH_2_OH in the gas phase have been made so far.

In this work, we present a computational analysis of several gas-phase reactive paths that can lead to NH_2_OH (and its cation), with particular attention to ion–molecule collision processes. This choice is motivated by the fact that ion–molecule collisions often do not present activation barriers along the entrance channel, and due to their typically large cross-sections, are more likely to proceed even at the low temperature and pressure of a molecular cloud.

In order to provide realistic production routes, we have considered reactions of the type:

(1) $$A+B\to {\mathrm{NH}}_{2}\mathrm{OH}+C$$

where the final partner C can carry away the excess energy due to the NH_2_OH formation. In this way, we overcome the problem of the reactions mentioned above, where the desired product is only a transient intermediate.

When dealing with charged systems, one must obviously consider the charge localization. In other words, one must consider the fact that both channels A^+^ + B and A + B^+^, depending on various constraints (e.g., multiplicity), can provide plausible reaction routes. This obviously poses a problem in the calculations, since only one of those channels corresponds to the ground state, while the other is an electronically excited state of the system. Therefore, common methodologies such as DFT or MP2 can only provide information about one of those states, while more sophisticated methods are required for the other.

In order to choose the reactants, we have relied on the most recent compendium of astrophysical molecules detected in space [19]. While the abundances of some neutral species are often known, the existence of the corresponding cations is not. In the case of reagents such as $H_{2}O_{2}^{+}$, ${\mathrm{NH}}_{2}^{+}$, and ${\mathrm{NH}}_{3}^{+}$, we must simply assume that given the existence of the parent neutral species, the chances of having their cations are not negligible. The presence of some cations has been instead directly inferred, either from observations or models, like for example, ${\mathrm{HO}}_{2}^{+}$, ${\mathrm{OH}}^{+}$, and $H_{2}O^{+}$ [20,21,22]. The relevant ionization energies of some of the species involved are reported in Table S1. These data clearly show the degree of confidence of the calculations methods here applied. One important issue to consider is that hydroxylamine has one of the lowest ionization potentials of all the species; hence, the ion–molecule reactions will tend, at least energetically, to favor the formation of its cation, rather than the neutral species.

## 2. Results

The reactions studied in this work are reported in Table 1 and include 19 ion–molecule reactions and two neutral ones (**20** and **21**). The data have been presented for two rather large basis sets (doubly polarized, triple and quadruple zeta) in order to show the convergences of the calculations, which are quite good in most cases. It is worth noting that some of the chemical species have multireference characters; hence, the CCSD(T) results (a single reference method) can be affected by small errors.

The last two reactions are indeed among those that can directly produce NH_2_OH in its neutral form, but both are hindered by large (>20 kcal/mol) activation barriers and are reported here only for completeness, being essentially kinetically impossible at low temperatures. These two reactions will not be further investigated here.

All but two of the other ion–molecule reactions listed in Table 1 are highly exoergic, thereby providing thermodynamically allowed reactive channels toward the formation of hydroxylamine or its cation NH_2_OH^+^. We explore these reactions in detail in the next sections.

### 2.1. The Reactions Involving NH_3_, OH, and Their Cations

The simplest chemical reactions that we have analyzed involve NH_3_ and OH, and their cations. The reactions evolve toward the release of an H atom or a proton.

**Figure 1:**
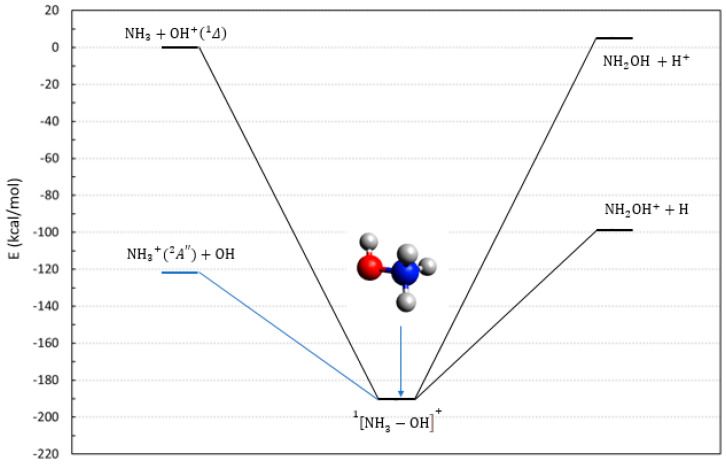
Energetic scheme of the possible entrance and exit channels of reactions **1** and **2**, involving the system [OH–NH_3_]^+^ in its singlet multiplicity. Energies computed at CCSD(T)/Def2-TZVPP level.

Reactions **1** and **2** have the same entrance channel with neutral NH_3_, evolve over a singlet potential energy surface (PES), and lead to a pair of doublet radicals. The entrance channel requires an electronically excited state of OH^+^ in a singlet spin state. A scheme summarizing the asymptotic energies in the singlet manifold is reported in Figure 1. Given that the ionization potential of hydrogen is larger than that of NH_2_OH, the path leading to neutral NH_2_OH (reaction **2**) must evolve on an excited potential energy surface and is slightly endothermic. Our calculation yielded a positive reaction energy of between 2–5 kcal/mol. However, this value is underestimated due to the single reference nature of the CCSD(T) method. With this method, we are able to locate the Δ1 state of OH^+^ 58.6 kcal/mol above the ground state (see Table S2), but the available experimental data [23] set this number to 49.8 kcal/mol. Hence, the energy needed by reaction **2** is very likely 10 kcal/mol larger than our estimate. For this reason, reaction **2** can be safely considered as endoergic, and is unable to be effective to form neutral hydroxylamine under astrophysical conditions.

As shown in Figure 1, there is a thermodynamically open channel toward NH_2_OH^+^ through reaction **1**. This looks to be a plausible route toward the formation of hydroxylamine cations, due to the large energy gain. The only limit to this path is due to the competitive charge exchange reaction:

(2) NH3+OH+Δ1→NH 3+A″2+OH 

whose gain in energy is around 120 kcal/mol, and whose products are the reactants of reaction **4**.

By looking at Figure 1, one can see that both routes to hydroxylamine (neutral and cation), starting with cationic ${\mathrm{NH}}_{3}^{+}$ (in blue in Figure 1) and passing through a singlet PES, are endothermic.

**Figure 2:**
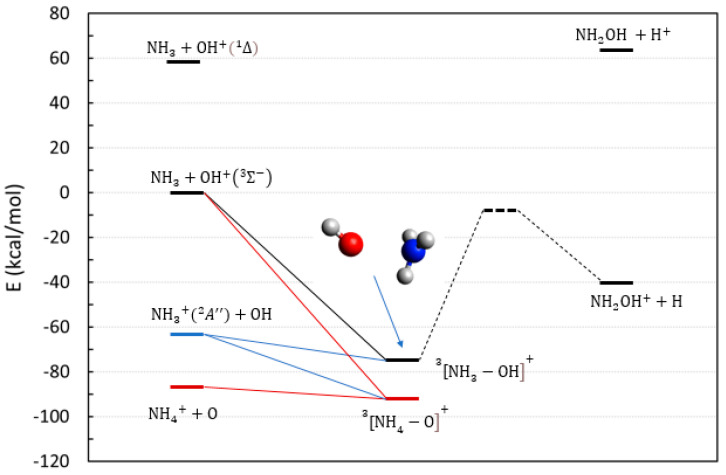
Energetic scheme of the possible entrance and exit channel of reactions **3** and **4** involving the system ${\left[{\mathrm{NH}}_{3}-\mathrm{OH}\right]}^{+}$ with triplet multiplicity. The relative energies of the reactants of reactions **1**–**2** in their singlet state (top left) are also shown for comparison. Energies computed at CCSD(T)/Def2-TZVPP level.

Reactions **3** and **4** represent two other routes toward hydroxylamine and its cation, and both evolve on a triplet PES. A scheme of the thermodynamic energies for these reactions is reported in Figure 2. Neutral hydroxylamine is thermodynamically inaccessible for both entrance channels, but an exothermic route is represented by the process of reaction **3** that leads to the hydroxylamine cation.

In this case, we have a ground state OH^+^ (a triplet) that reacts with a closed shell ammonia molecule. The reaction obviously evolves on a triplet potential energy surface and yields two doublet radicals, one being an H atom. However, such a reaction reveals itself to be ineffective when explored at the CASSCF/NEVPT2 level. The relevant data are reported in Figure S1, where a calculation of the first five electronic states of the [NH_3_–OH]^+^ complex is presented, along the variation of the N–O distance. The potential energy curve of triplet multiplicity correlating with the NH3+OH+Σ3− channel is repulsive at short distances, thereby preventing the formation of the N–O bond, and hence, the existence of a corresponding low-energy ^3^[NH_3_–OH]^+^ complex that is able to evolve toward the products.

Reaction **4**, starting from NH3+A″2+OH, is endothermic at ~20 kcal/mol and is apparently not worth investigating. However, an analysis of the fate of the initial ^3^[NH_3_–OH]^+^ complex (whose stable geometry is illustrated in Figure 2) might still be useful because it could also be formed by the (radiative or non-radiative) electronic decay of the reactants of reaction **3**. We were able to characterize two reactive paths, bringing the ^3^[NH_3_–OH]^+^ complex into the final cationic hydroxylamine, and as expected, they both show large barriers ranging from 65 to 80 kcal/mol due to the breaking of either an O–H or an N–H bond. The energies and the geometries of these two reaction paths are reported in Figures S2 and S3. The presence of these barriers is generically indicated by the dotted lines in Figure 2. One of these reactive paths ends with the hydroxylamine structural isomer NH_3_O^+^. This isomer can interconvert to the hydroxylamine cation, but the process requires 32.6 kcal/mol, as shown by additional calculations reported in Figure S4.

In addition to the path leading to the hydroxylamine cation, there is another parasitic and thermodynamically open channel that leads to the stable products ${\mathrm{NH}}_{4}^{+}$, and a neutral oxygen atom (red lines in Figure 2). This is a simple proton exchange, and as such, it is a barrierless reaction, thus further casting a strict limit to the efficiency of reaction **3** to proceed toward the hydroxylamine cation.

In conclusion, we have explored various possible reaction pathways toward hydroxylamine, starting from the simplest ingredients (OH and NH_3_). Among the reactions presented, the only ones that are thermodynamically accessible under astrochemical conditions lead to NH_2_OH^+^. Those that could be taken into consideration are reaction **1,** where an electronically excited state of OH^+^ is involved, and reaction **3**, where a transition between two triplet electronic states is required. The latter process could be summarized as:

(3) NH3+OH+Σ−3→NH3−OH+*3→NH3−OH+3→NH2OH++H

where, due to the initial high energy content of the reactants (~60 kcal/mol above the ground state), it might be possible to overcome the reaction barriers leading to hydroxylamine indicated in Figure 2. However, this process is hampered by a parasitic chemistry producing a more energetically favorable ammonium cation. Overall, we can conclude that the reactions involving ammonia and the OH moiety seems to be highly ineffective in forming hydroxylamine.

### 2.2. The Reactions Involving Water and Its Cation

**Figure 3:**
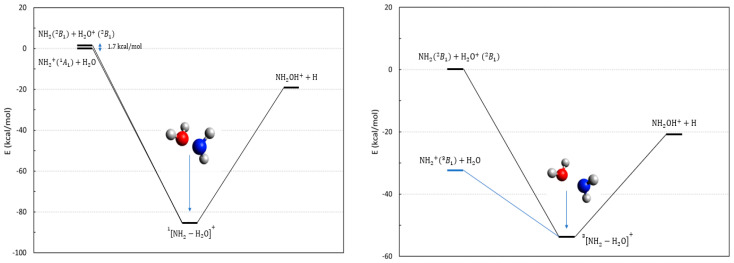
Energetic scheme of the possible entrance and exit channel of reactions **5** and **6** involving the system ${\left[{\mathrm{NH}}_{2}-H_{2}O\right]}^{+}$ with singlet (**left**) and triplet (**right**) multiplicity. The blue line in the right panel is the ground state entrance channel. Energies computed at CCSD(T)/Def2-TZVPP level.

Reactions **5** and **6** are two exothermic processes involving the amino moiety NH_2_/${\mathrm{NH}}_{2}^{+}$ and water (cationic or neutral). A scheme of the possible thermodynamical channels is reported in Figure 3 for both singlet and triplet multiplicities. In the same figure, we have reported the geometries of the reaction complexes for the triplet and singlet cases. Their geometries are very similar, but the triplet complex is less stable with respect to the entrance channel (~55 kcal/mol vs. 83 kcal/mol), and it has a larger N–O distance.

The reaction involving the ground state entrance channel has a triplet multiplicity and would be:

(4) NH2+B13+H2O→NH2OH++H

where both reactants are in their electronic ground state. This reaction is endoergic, at about 12 kcal/mol, it does not constitute a viable formation channel for the hydroxylamine cation, and it is not listed in Table 1, but its energetic location is nevertheless shown in Figure 3 (blue line in the right panel).

The charge exchange state of the entrance channel of reaction (4) corresponds to the two ground state radicals NH2B12+H2O+B12 in a global singlet or triplet state that can evolve accordingly to reaction **5**. Another possible entrance channel involves the first electronic excited state of ${\mathrm{NH}}_{2}^{+}$, and it is represented by the collision of reaction **6**. This channel is only 1.7 kcal/mol below the previous one.

Reaction **5** is exoergic at about 21 kcal/mol, is thermodynamically accessible under astrochemical conditions, and can proceed toward the hydroxylamine cation through both a singlet and a triplet PES; however, its evolution in both cases is hindered by the appearance of a repulsive potential due to an increase in electronic energy upon compressing the N–O distance. Detailed energies at the CASSCF/NEVPT2 level are reported in Figures S5 and S6 (respectively, for singlet and triplet cases) where we show the energies of the first four electronic states in the entrance channel and follow them through a possible reactive path toward the products. Reaction **6** involving neutral water is exoergic toward the hydroxylamine cation, and as shown in Figure S5 (blue curve), presents a downhill path without any barrier, hence representing a viable formation channel of the hydroxylamine cation. The entrance channel of reaction **5** instead do not seem to correlate with the hydroxylamine cation, but only with its neutral form, hence leading to a process taking place on a repulsive, endoergic PES (Figures S5 and S6, orange curve). In other words, reaction **5** appears impossible, at least on a single PES.

In conclusion, using the presented data, we can say that a collision between the ground state ${\mathrm{NH}}_{2}$ and $H_{2}O^{+}$ (reaction **5**) in a global triplet state can only produce a charge exchange leading back to ${\mathrm{NH}}_{2}^{+}$ and $H_{2}O$ (which is the ground state). The same collision (again, reaction **5**) in a global singlet state appears to be non-reactive, or at least, quite ineffective due to a repulsive PES.

In conclusion, the only reactive process that can form a hydroxylamine cation seems to be reaction **6,** which, although requiring an excited state of ${\mathrm{NH}}_{2}^{+}$, is barrierless and probably efficient. We cannot exclude that due to the energetic proximity of the respective electronic states, the collision of ${\mathrm{NH}}_{2}$ and $H_{2}O^{+}$ in a singlet state can interconvert into NH2+A 11 and $H_{2}O$, thus leading to the hydroxylamine cation via the same PES of reaction **6**. Thus, we can propose a second possible mechanism toward a hydroxylamine cation that would start from two ground state molecules and proceed along two singlet PESs, as:

(5) $${\mathrm{NH}}_{2}+H_{2}O^{+}\to {\left({\mathrm{NH}}_{2}^{+}\right)}^{*}+H_{2}O\to {\mathrm{NH}}_{2}{\mathrm{OH}}^{+}+H$$

### 2.3. The Reactions Involving OOH, the Hydroperoxyl Radical

Reactions **7**–**13** all involve the hydroperoxyl radical or its cation. The ground state of OOH is a doublet, and that of OOH^+^ is a triplet. The latter has a singlet excited state just 0.3 eV (~7 kcal/mol) above the lowest triplet one (see Table S2). Reactions **7**–**10** involve either NH_2_ in its ground state (B 21) or ${\mathrm{NH}}_{2}^{+}$ in its ground (B13) and first excited state (A11). Reactions **11**–**13** involve the ammonia molecule or its cation in their ground states.

**Figure 4:**
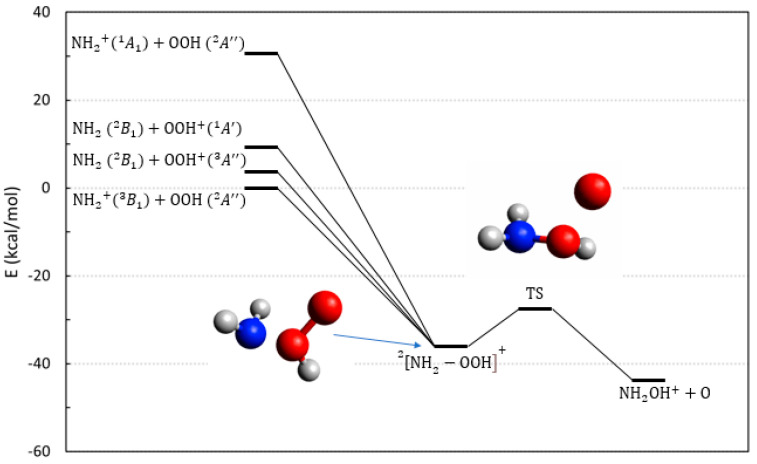
Energetic scheme of the possible entrance and exit channel of reactions **7**–**10** involving the system ${\left[{\mathrm{NH}}_{2}-O_{2}H\right]}^{+}$ with doublet multiplicity. The structure of the ground state intermediate complex and that of the transition state toward the product (O–O bond break) are also shown. Energies computed at CCSD(T)/Def2-TZVPP level.

The thermodynamics of reactions **7**–**10**, all in their doublet multiplicities, are shown in Figure 4. Due to the ionization potential of the oxygen atom being 4.5 eV larger than that of hydroxylamine, the exit channel involving O^+^ is more than 100 kcal/mol above the one with neutral O. In addition, the overall multiplicity constrains the (doublet) force, considering only those reactions that end with neutral O (triplet), plus the hydroxylamine cation (a radical doublet).

All reactions are exoergic due to the instability of the hydroperoxyl, and can proceed either on doublet or quartet PESs, but we have limited our analysis to doublet multiplicity. The ground state (reaction **9**) initiates with ${\mathrm{NH}}_{2}^{+}$ (triplet) plus OOH (doublet); the reaction forms a stable complex (See Figure 4) and evolves toward the final product by breaking the O–O bond. This generates a barrier along the exit channel that is below the initial energy. Due to the system size, we were not able to perform CASSCF calculations using the entire valence active space, and our attempts to select a subset of valence orbitals invariably finished with the failure to describe, at the same time, the four entrance channels. We have been able to obtain only qualitative results that indicate that the PES for reaction **10** is repulsive and ineffective. The evolution for reactions **7**, **8**, and **9** cannot be traced with the same certainty because the three PES are strongly coupled in the entrance channel, with several crossings appearing before the intermediate complex of Figure 4. Due to this, we can only surmise that all of them are probably able to evolve toward the products. In addition, these processes can also evolve toward the final release of an excited oxygen atom O(^1^D) which is asymptotically located 45 kcal/mol above the products of Figure 4, hence, significantly reducing or erasing altogether the energetic gain of the reaction. Finally, we cannot consider these reactions as being efficient channels toward NH_2_OH^+^, because of the competitive proton transfer process:

(6) $${\mathrm{NH}}_{2}+{\mathrm{OOH}}^{+}\to {\mathrm{NH}}_{3}{}^{+}+O_{2}$$

that is exoergic (−84.5 kcal/mol) and barrierless.

**Figure 5:**
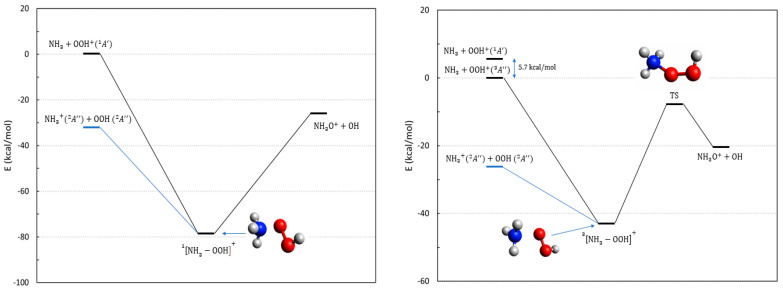
Energetic scheme of the possible entrance and exit channels of reactions **11**–**13** involving the system ${\left[{\mathrm{NH}}_{3}-O_{2}H\right]}^{+}$ with singlet (**left**) and triplet (**right**) multiplicity. The blue line in both panels is the ground state. Energies computed at CCSD(T)/Def2-TZVPP level. The energy of the singlet entrance channel NH3+OOH+A′ 1 is also reported in the right panel for clarity.

Reactions **11**–**13** have been identified as possible reactive paths, starting from a collision between ammonia and hydroperoxyl. Different from the previous peroxide processes, these can evolve on either a triplet or a singlet PES. The relevant thermodynamics are illustrated by the energies in Figure 5. Due to the low ionization potential of ammonia, the ground state entrance channel is made by two doublets, ${\mathrm{NH}}_{3}^{+}$ and OOH, both in their ground states.

Reaction **13** is the one pertaining to the ground state and can proceed through both a singlet and triplet intermediate whose stable geometries are reported in Figure 5. Both reactions can then only proceed toward the hydroxylamine isomer ${\mathrm{NH}}_{3}O^{+}$ and are endoergic. In the case of the triplet multiplicity, the ground state in the exit channel shows the appearance of a barrier before the formation of ${\mathrm{NH}}_{3}O^{+}$ (Figure 5, right panel). In addition, as shown in Figure S4, the ensuing conversion of ${\mathrm{NH}}_{3}O^{+}$ to NH_2_OH^+^ would require passing through a barrier of 36.2 kcal/mol to obtain the final product (with the net gain in energy reported in Table 1). Hence, we conclude that reaction **13** is not a possible route to hydroxylamine in astrochemical conditions.

Reactions **11** and **12** with OOH^+^, respectively, in a triplet and a singlet state, are thermodynamically open due to exothermicity, and they may provide additional routes toward hydroxylamine cation. Their efficiency, however, is limited because of the appearance of the proton transfer reaction channel:

(7) $${\mathrm{NH}}_{3}+{\mathrm{OOH}}^{+}\left(A^{\prime \prime }\right)\to {\mathrm{NH}}_{4}^{+}+O_{2}$$

that is highly exoergic (−102.4 kcal/mol) and free from barriers.

In conclusion we have explored seven different reactions involving hydroperoxyl radical that could possibly lead to the hydroxylamine cation. Some of them (reactions **10** and **13**) are ineffective due to repulsive PESs or to the appearance of barriers in the exit channels; the rest, even though probably viable, suffer from the competition of efficient proton transfer reactions with larger exothermicities, and are free from activation barriers.

### 2.4. The Reactions Involving the H_2_O_2_ Peroxide

**Figure 6:**
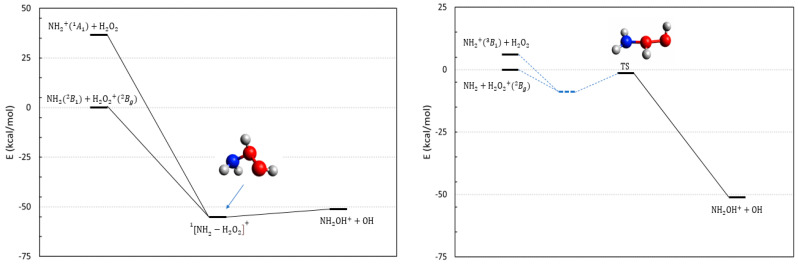
Energetic scheme of the possible entrance and exit channel of reactions **14**–**16** involving the system ${\left[{\mathrm{NH}}_{2}-H_{2}O_{2}\right]}^{+}$ with singlet (**left**) and triplet (**right**) multiplicity. Energies computed at CCSD(T)/Def2-TZVPP level. The intermediate complex for the singlet case is shown. In the right panel, we have reported the only stationary point that we have been able to locate that corresponds to a transition state.

Reactions **14**–**16** involve hydrogen peroxide and its cation. H_2_O_2_ is the smallest chiral molecule, owing to the asymmetry of its anti conformation that is the most stable one [24]. Its cation is a radical in a doublet state. Since ionizing H_2_O_2_ is easier than NH_2_, the ground state of the system corresponds to the entrance channel of reaction **14**. The energetics of the reactive channels are illustrated in Figure 6. This reaction can proceed on a singlet (left panel in Figure 6) or triplet (right panel of Figure 6) PES, and in both cases, is exoergic. While for the singlet PES we have been able to locate the reaction complex, its equivalent for the triplet PES has eluded our search.

Slightly above the ground state, we have located the triplet charge exchange state (the reactants of reaction **15**), and at even higher energies, we find the reactants of reaction **16** that involve an excited state of ${\mathrm{NH}}_{2}^{+}$.

Qualitative results at the CASSCF/NEVPT2 level (reported in Figure S7) show that reaction **14** in its singlet state is free of barriers and proceeds directly toward the hydroxylamine cation. Reaction **16** on the other hand, takes place on a repulsive PES.

Overall, the collision between the two ground state radicals NH_2_ and $H_{2}O_{2}^{+}$, either in a global singlet or triplet state, seems to be a possible path toward NH_2_OH^+^, although the latter is hindered by a significant barrier in the exit channel whose transition state possesses an energy that is almost equal to the reactants (Figure 6). As for other reactions, such processes can suffer the competition of other exoergic channels such as the proton transfer reaction:

(8) NH2 B12+H2O2+→NH3++OOH

### 2.5. The Reactions Involving Nitrous Acid, HONO

The detection of nitrous [5] acid in the ISM (albeit not in the G+0693 molecular cloud) has led us to explore reactive processes involving it. Nitrous acid is a reactive species and can act as a hydrogen donor toward smaller molecules. Its ion could be formed by proton transfer to NO_2_ by relative abundant proton donors such as $H_{3}^{+}$. The presence of HONO^+^ has been therefore hypothesized to be plausible.

We have computed the thermochemistry of three reactions involving HONO. Reactions **17**–**18** can proceed through a collision between the HONO^+^ and the NH_2_ radical; they are essentially the same reaction that evolves on the ground (singlet and triplet) PES of the system. The two reactions differ only for the products that are similar in energy, with a difference of only 0.4 kcal/mol. When reaction **17** evolves with a global triplet multiplicity, owing to spin conservation, this can only lead to the hydroxylamine cation and neutral NO (radical). The reaction is barrierless and the system moves on a PES that is purely downhill toward the products. The same reaction could, in principle, also proceed on a global singlet PES, but the PES is repulsive.

Reaction **18** can only take place in a global singlet state, and remarkably, it is the only exoergic process in Table 1 that can lead directly to neutral hydroxylamine. The reaction can proceed through two different paths that have been outlined in Figure S8. In both cases, the reaction profile was free from barriers, and as proven through additional calculations (Figure S9), one of the two intermediate complexes can evolve adiabatically toward neutral hydroxylamine.

Reaction **19** evolves along the first excited state of the system and corresponds to the charge exchange entrance channel with respect to the previous two. By performing a scan along the N–O distance in the entrance channel at the CASSCF/NVPT2 level, we have detected that reaction **19** is ineffective since the relative PES is strongly repulsive.

In conclusion, we have identified a direct barrierless process (reaction **18**) that involves the amino radical and the nitrous acid cation. The efficiency of the process is obviously due to the abundance of HONO^+^, which now is yet undetected, while its parent neutral is known to exist. Additional limits to the likelihood of the process come from the fragility of the nitrous cation [25] that is prone to fragmentation.

Additional constraints on the importance of reaction **18** come from the presence of parasitic chemistries arising from other exothermic dissociation patterns of the reaction complexes in Figure S8. Particularly, both the following reactions are exoergic, at −44 kcal/mol and −114 kcal/mol, respectively.

(9) $${\mathrm{HONO}}^{+}+{\mathrm{NH}}_{2}\to {\mathrm{NO}}_{2}+{\mathrm{NH}}_{3}^{+}$$

(10) $${\mathrm{HONO}}^{+}+{\mathrm{NH}}_{2}\to N_{2}O+H_{3}O^{+}$$

The first is a simple, typically barrierless, proton transfer, while the second requires the formation of a new N–N bond whose steps are illustrated in Figure S10.

## 3. Methods

The calculations have been performed using the ORCA code (versions 5.03) [26]. The geometries of all molecules have been obtained via either the MP2 method [27] or the B3LYP-D3BJ functional. All products and reactants minimum energy geometries have been characterized using a frequency calculation that has also allowed for the evaluation of the harmonic ZPE (zero-point energy) corrections to the electronic energy at the MP2/DFT level. The final energies have been evaluated using the CCSD(T) method at the MP2 or DFT geometry [28]. The methods chosen can be considered as a sufficiently accurate, standard approach that allows for reliable geometries and electronic energies to be obtained (e.g., see analogous calculations in refs. [29,30,31]).

Since in the astrochemical condition of a molecular cloud the internal degrees of freedom of the molecules are not obviously thermalized or canonical, we avoid presenting thermodynamic functions, and we shall limit ourselves to the CCSD(T) electronic energies (combined with the vibrational ZPE, as obtained using the MP2 frequencies). We point out that sometimes this recipe can lead to a slight overestimation of the ZPE contribution, due to a known problem with the single reference nature of the MP2 method, especially in open-shell systems [32]. The basis sets employed are the triple-zeta (doubly polarized) def2-TZVPP for geometries and energies, and its quadruple zeta counterpart def2-QZVPP for energies only. As one can see from the data in Table 1, the use of a polarized triple-zeta basis is essentially sufficient to converge the results. When excited state calculations were needed, we used CASSCF calculations with the def2-TZVPP basis within suitable active spaces whose dimension ranges from (12,10) to (14,12), depending on the specific molecular system. The CASSCF calculations essentially consisted of the optimization of the interacting complex, and in a series of “relaxed” scans along one or more coordinates, to obtain an energy profile toward the reactants and products. The CASSCF energy values were correlated via a NEVPT2 perturbative scheme [33,34].

## 4. Summary and Conclusions

We have explored and presented a rather long list of possible gas-phase reactions whose outcome is either hydroxylamine or its cation. Hydroxylamine is an important pre-biotic molecule, together with molecules such as urea, ethanolaminem, and propylene oxide [35] (the only chiral molecule of the batch), whose detection shows how interstellar medium chemistry can bear a complex oxygen–nitrogen chemistry that is the necessary prelude to biochemistry [10].

A recent work [14] has shown how hydroxylamine can be formed through the hydrogenation of NO, owing to the catalytic action of icy dust grains. However, we were curious to investigate whether gas-phase chemistry could also contribute to its formation. Overall, the answer is probably negative. Only one process among the many explored here is directly able to form neutral hydroxylamine through a barrierless, ion–molecule reaction. This is the process identified by reaction **18**, where a nitrous acid [36] cation reacts with an amino radical. [37] However, in addition to the presence of competitive exoergic channels, the reaction is probably doomed to be quite inefficient due to being controlled by the abundance of ionized nitrous acid, as yet an undetected and fragile species susceptible to facile dissociation.

There are, however, a certain amount of ion–molecule collision processes whose likely outcome is ionized hydroxylamine. For example, reaction **6** could be a viable route, but it requires an excited state (singlet) of the amino radical. This process could, however, be involved in a more complicated sequence such as that of Equation (5), where the excited amino radical is formed through potential energy surface crossing in a collision between ground state water and ${\mathrm{NH}}_{2}^{+}$.

A collision between the two ground state radicals NH_2_ and $H_{2}O_{2}^{+}$, either in a global singlet or a triplet state, is also able to produce the hydroxylamine cation through exoergic processes (reaction **14**). We point out, however, that it has to compete with other exoergic processes (e.g., Equations (7)–(10)) that involve simple proton transfers that are typically free from activation barriers.

In conclusion, thanks to this survey, we believe that the direct formation of hydroxylamine in the interstellar medium via gas-phase chemistry is a possible but highly unlikely event. The formation of its cation is much more probable, but the efficiency of its neutralization via charge exchange with other neutral species (electron capture would be dissociative) is unexplored at the moment. Given the present analysis, it is plausible to assume that the detected hydroxylamine comes from grain chemistry, and eventually, its thermic or mechanical desorption.

## Figures and Tables

**Table 1:** List of the reactions examined in this work, along with their reaction energies (kcal/mol), corrected for ZPE. Geometries and ZPEs are calculated at the MP2 or DFT level. Electronic energies are at the CCSDT(T) level. When not obvious, the term symbol of the molecular species has been indicated.

Reaction	2s + 1	Def2-TZVPP	Def2-QZVPP
**1** NH3+OH+Δ 1→NH2OH++H	1	−98.9	−99.8
**2** NH3+OH+Δ 1→NH2OH+H+	1	+5.0	+2.2
**3** NH3+OH+Σ 3−→NH2OH++H	3	−40.3	−41.8
**4** NH3+A″2+OH →NH2OH++H	3	+22.9	+22.5
**5** NH2B 21+H2O+B 21→NH2OH++H	1/3	−20.8	−21.8
**6** NH2+A 11+H2O→NH2OH++H	1	−19.1	−21.7
**7** NH2B 21+OOH+(A″3)→NH2OH++O	2/4	−47.4	-
**8** NH2B21+OOH+A′1→NH2OH++O	2	−53.1	−52.5
**9** NH2+B31+OOHA″2→NH2OH++O	2/4	−43.7	-
**10** NH2+A11+OOH A″2→NH2OH++O	2	−74.3	−73.6
**11** NH3+OOH+A″3→NH2OH++OH	3	−41.8	-
**12** NH3+OOH+A′1→NH2OH++OH	1	−47.5	−47.5
**13** NH3+A″2+OOHA″ 2→NH2OH++OH	1/3	−15.5	−25.0
**14** NH2B21+H2O2+B2g→NH2OH++OH	1/3	−51.2	−48.5
**15** NH2+B31+H2O2→NH2OH++OH	3	−57.2	-
**16** NH2+A11+H2O2→NH2OH++OH	1	−87.8	−88.7
**17** HONO+A′2+NH2B21→NH2OH++NOΠ2	1/3	-	−59.5
**18** HONO+A′2+NH2B21→NH2OH+NO+Σ1+	1	-	−59.9
**19** HONOA′1+NH2+B31→NH2OH++NOΠ2	3	-	−58.0
**20** NH2B21+OOHA″2→NH2OH+O	3	+0.3 ^a^	+0.8
**21** NH2B21+H2O2→NH2OH+OH	2	−13.3 ^a^	−13.7

^a^ Both reactions are characterized by large activation barriers above 20 kcal/mol.

## Data Availability

The data presented in this study are available upon request from the corresponding author.

## Supplementary Materials

The following supporting information can be downloaded at: https://www.mdpi.com/article/10.3390/molecules28072932/s1, Table S1: Adiabatic first ionization energies (eV) computed at the MP2//CCSD(T)/Def2-TZVPP level. Table S2: Singlet-triplet separation energies (eV) at the MP2//CCSD(T)/Def2-TZVPP level. Figure S1: Energy scan along the N-O coordinates of the first 6 electronic states of the [NH_3_-OH]^+^ system. Figure S2: Reactive paths starting from ${\mathrm{NH}}_{3}^{+}+\mathrm{OH}$ on the triplet PES. Figure S3: Alternative reactive path starting from ${\mathrm{NH}}_{3}^{+}+\mathrm{OH}$ on a triplet PES. Figure S4: Isomerization minimum energy paths and energies for NH_3_O and NH_3_O^+^ to hydroxylamine and its cation. Figure S5: Energies of the relevant electronic states along the entrance channels of reaction **5** (orange color) and **6** (blue color) for a singlet multiplicity. Figure S6: Relevant electronic states along a possible path for reaction **5** (orange color) for triplet multiplicity. Figure S7: Relevant electronic states along a possible path for reaction **14** (blue color) in a singlet multiplicity. Figure S8: Thermodynamics for reaction **18**. The data are computed for a global singlet state. Figure S9: Electronic energy of the exit channel of reaction **18.** Figure S10: Alternative reactive path starting from ${\mathrm{HONO}}^{+}+{\mathrm{NH}}_{2}\;$ on a singlet PES. Refs. [38,39,40,41,42] are cited in the Supplementary Materials.
