# Supplementary material for: About the Formation of NH2OH+ from Gas Phase Reactions under Astrochemical Conditions
Source: Molecules. 2023 Mar 24;28(7):2932. doi: 10.3390/molecules28072932 (PMC10096285; doi:10.3390/molecules28072932)
Supplement: Supplementary file 1 [file molecules-28-02932-s001.zip › molecules-2286286-supplementary.pdf]

Formation of NH_2OH^+ from gas phase reactions and its relevance for the chemistry of the interstellar medium.

Gabriele Dilena[1], Simone Pistillo, and Enrico Bodo [1,*]

Supporting Information

Table S1: Adiabatic first ionization energies (eV) computed at the MP2//CCSD(T)/Def2-TZVPP level.

	This work	Literature
$\mathbf{NH_2OH}$	9.10	-
$\mathbf{NH_2}$	11.00	11.46 [a]
$\mathbf{NH_3}$	10.02	10.175 [b]
$\mathbf{H_2O}$	12.40	12.62 [c]
$\mathbf{OH}$	12.77	13.01 [d]
$\mathbf{HO_2}$	11.17	11.35 [e]
$\mathbf{H_2O_2}$	10.74	10.62 [f]

a) S. J. Dunlavey, J. M. Dyke, N. Jonathan, and A. Morris, Mol. Phys., 39, 1121–1135, 1980.
b) M. J. Weiss and G. M. Lawrence, J. Chem. Phys., 53, 214–218, 1970.
c) C. R. Brundle and D. W. Turner, Proc. R. Soc. London, 307, 27–36, 1968.
d) H. Van Lonkhuyzen and C. A. De Lange, Mol. Phys., 51, 1984.
e) J. M. Dyke, N. B. H. Jonathan, A. Morris, and M. J. Winter, Mol. Phys., 44, 1981.
f) F. S. Ashmore and A. R. Burgess, J. Chem. Soc. Faraday Trans. 73, 7, 1977.

Table S2: Singlet-triplet separation energies (eV) at the MP2//CCSD(T)/Def2-TZVPP level.

	This work	Literature
$\mathbf{NH_2}^+(^3\boldsymbol{B_1}) \rightarrow \mathbf{NH_2}^+(^1\boldsymbol{A_1})$	1.33	0.99 [a]
$\mathbf{OOH}^+(^3\mathbf{A}'') \rightarrow \mathbf{OOH}^+(^1\boldsymbol{A}')$	0.25	0.30 [b]
$\mathbf{OH}^+(^3\boldsymbol{\Sigma}^-) \rightarrow \mathbf{OH}^+(^1\boldsymbol{\Delta})$	2.54	2.16 [c]

a) S. J. Dunlavey, J. M. Dyke, N. Jonathan, and A. Morris, Mol. Phys., 39, 1121–1135, 1980.
b) J. M. Dyke, N. B. H. Jonathan, A. Morris, and M. J. Winter, Mol. Phys., 44, 1981.
c) H. Van Lonkhuyzen and C. A. De Lange, Mol. Phys., 51, 1984.

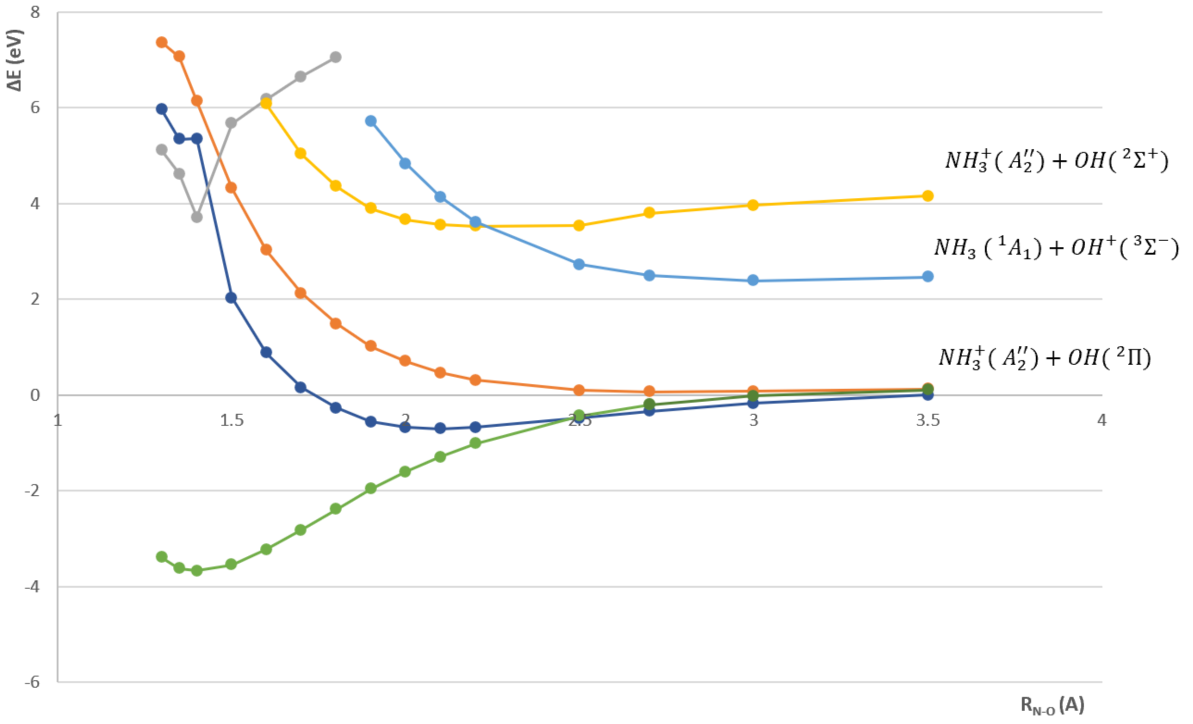

Figure S1: Energy scan along the N-O coordinates of the first 6 electronic states of the $[NH_3\text{-}OH]^+$ system. The green and orange curves are singlets, the other states are triplets. The yellow and gray curves are associated to excited states of neutral OH and are of no interest here. The cyan curve is instead important for the discussion and shows how the entrance channel of reaction **3** is repulsive toward the formation of the N-O bond. Two of the lowest energy curves correlating with NH_3^+ (blue and green, triplet and singlet respectively) have an attractive component but all paths emerging from them toward NH_2OH or its cation are endothermic (e.g. reaction **4** of Table 1). The data have been obtained using a CASSCF(14,12)/def2-TZVPP plus NEVPT2 correlation. The active space includes all valence orbitals and electrons.

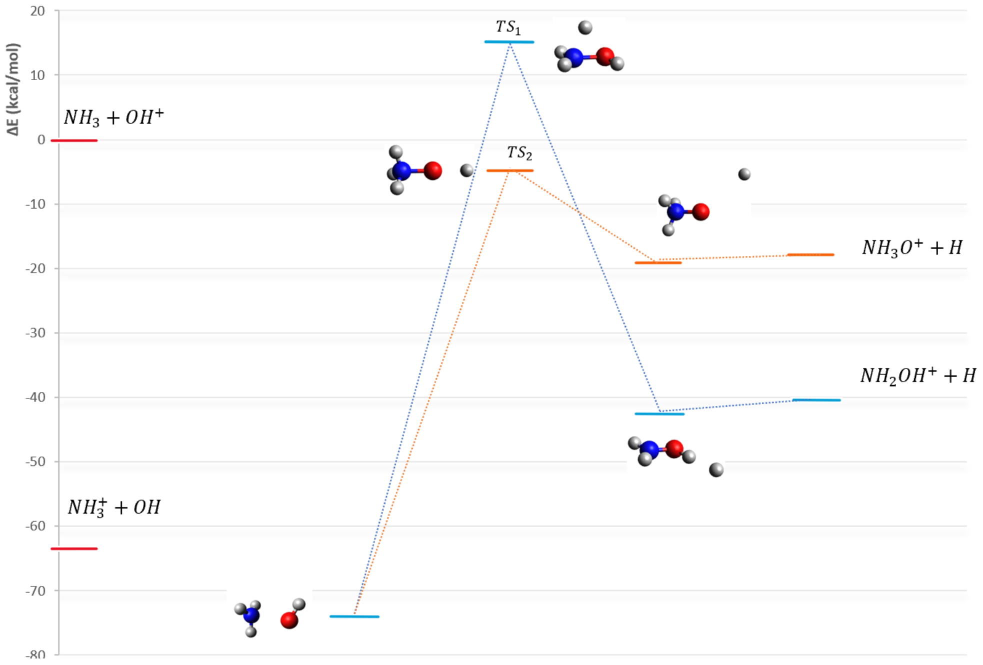

Figure S2: Reactive paths starting from $NH_3^+ + OH$ on the triplet PES. Starting from the $[NH_3 - OH]^+$ complex (bottom left) either PESs are repulsive, and the reaction proceeds through the breaking of the OH bond that requires ~70-90 kcal/mol depending on the final product: hydroxylamine cation or its isomer NH_3O^+. The energies have been computed at the B3LYP/def2-TZVPP//CCSD(T)/def2-QZVPP level.

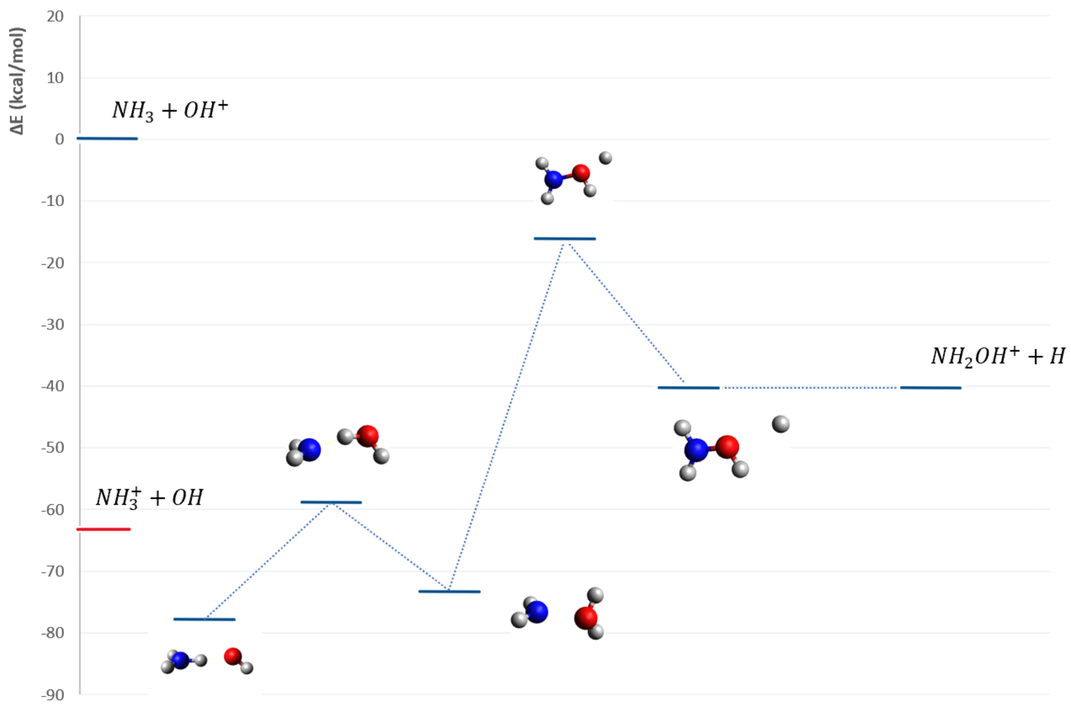

Figure S3: Alternative reactive path starting from $NH_3^+ + OH$ on a triplet PES. Starting from the $[NH_3 - OH]^+$ complex (bottom left) the PES is initially repulsive, the reaction proceeds through various steps, but the breaking of the OH bond requires ~65 kcal/mol. The energies have been computed at the B3LYP/def2-TZVPP//CCSD(T)/def2-QZVPP level.

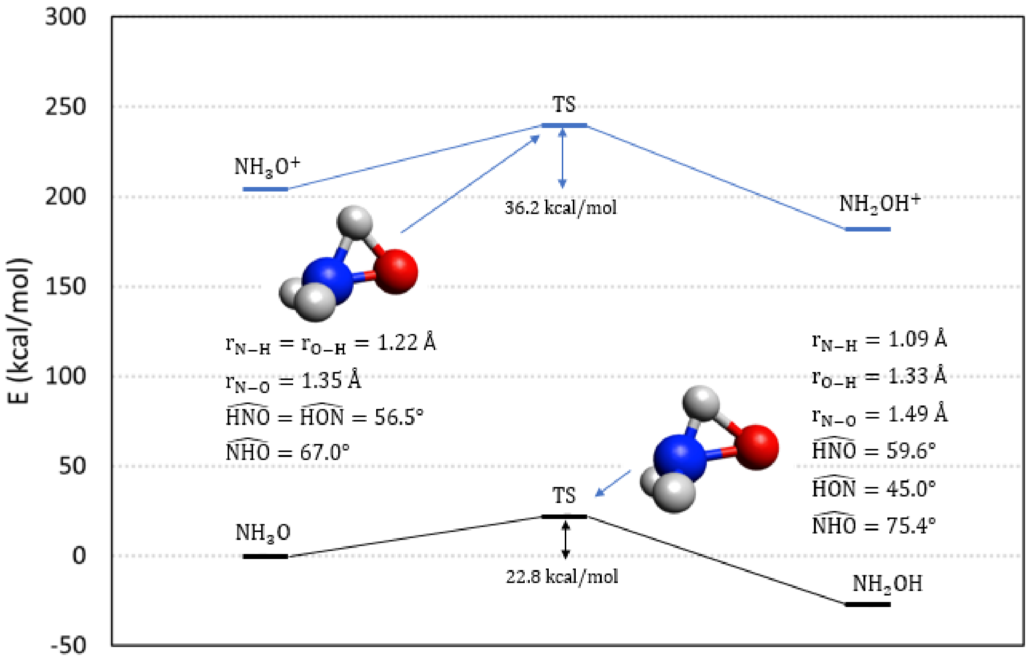

Figure S4: Isomerization minimum energy paths and energies for NH_3O and NH_3O^+ to hydroxylamine and its cation. The data have been obtained at the MP2//CCSD(T)/def2-TZVPP level.

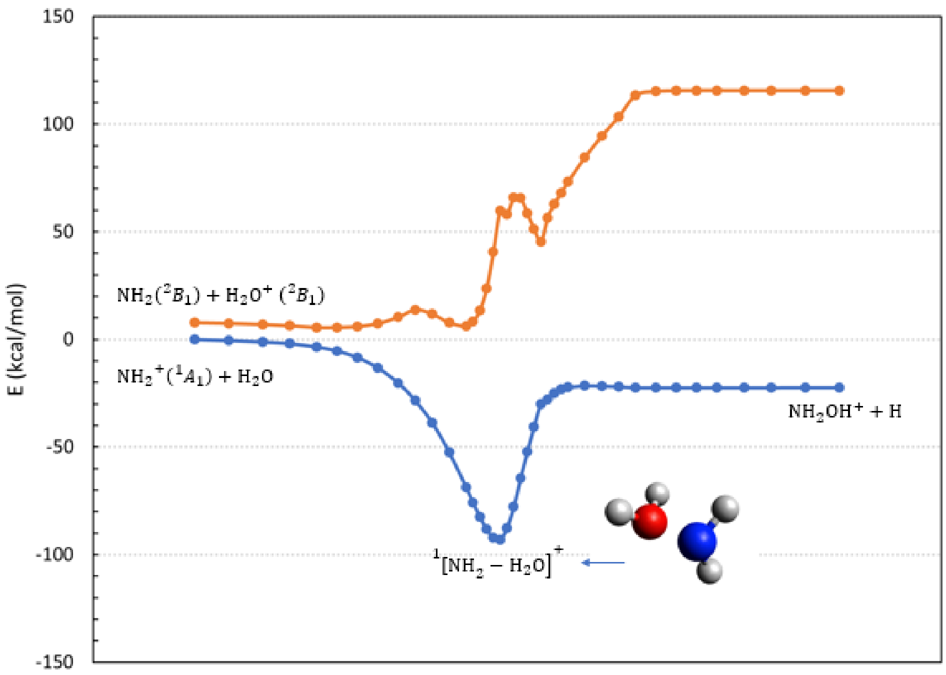

Figure S5: Energies of the relevant electronic states along the entrance channels of reaction **5** (orange color) and **6** (blue color) for a singlet multiplicity. The geometry of the ground state complex is also displayed. Before the complex the varying distance is N-O, after it, O-H. The energies have been evaluated using a CASSCF(14,12)/def2-TZVPP plus NEVPT2 correlation. The orange energies show a repulsive profile, highlight the absence of a stable complex and adiabatically correlate to neutral hydroxylamine plus a proton through a highly endoergic process. The blue energies represent instead a direct, barrierless path to hydroxylamine cation. The active space includes all valence orbitals and electrons.

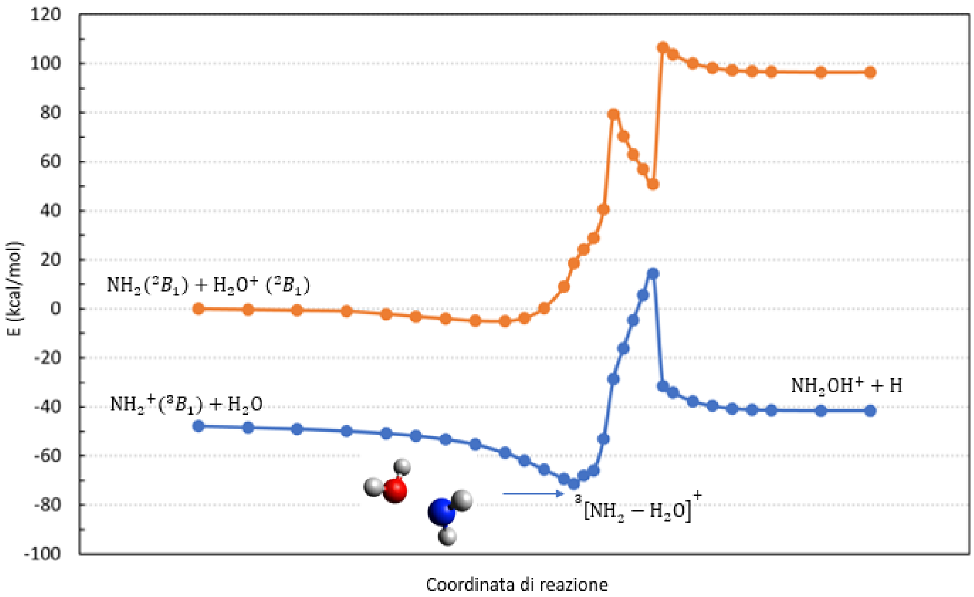

Figure S6: Relevant electronic states along a possible path for reaction **5** (orange color) for triplet multiplicity. The geometry of the ground state complex is also displayed. Before the complex the varying distance is N-O, after it, O-H. The blue curve in this case is the endoergic ground state reaction. The final products pertaining to the orange energies involve neutral hydroxylamine. Both PESs shows various discontinuities due to crossing other electronic states. The energies have been evaluated using a CASSCF(14,12)/def2-TZVPP plus NEVPT2 correlation. The active space includes all valence orbitals and electrons.

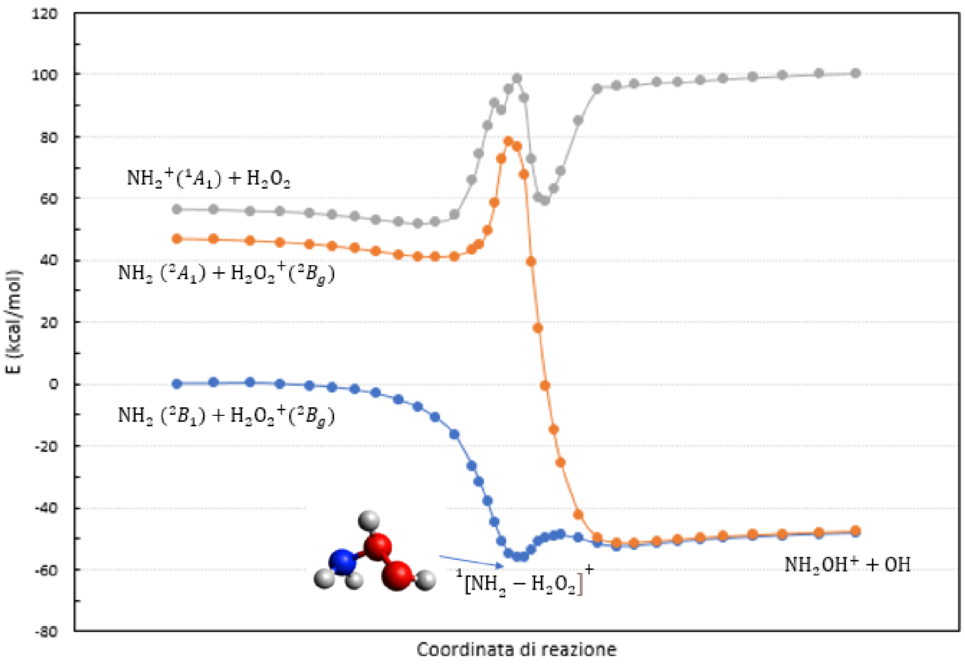

Figure S7: Relevant electronic states along a possible path for reaction **14** (blue color) in a singlet multiplicity. The geometry of the ground state complex is also displayed. Before the complex the varying distance is N-O, after it, O-O. The gray curve is the PES correlating with the reactants of reaction **16.** The energies have been evaluated using CASSCF(12,10)/def2-TZVPP plus NEVPT2 correlation. The active space is not complete (it does not include the lowest-lying valence orbitals) and the energies of the excited states, should be considered only qualitative.

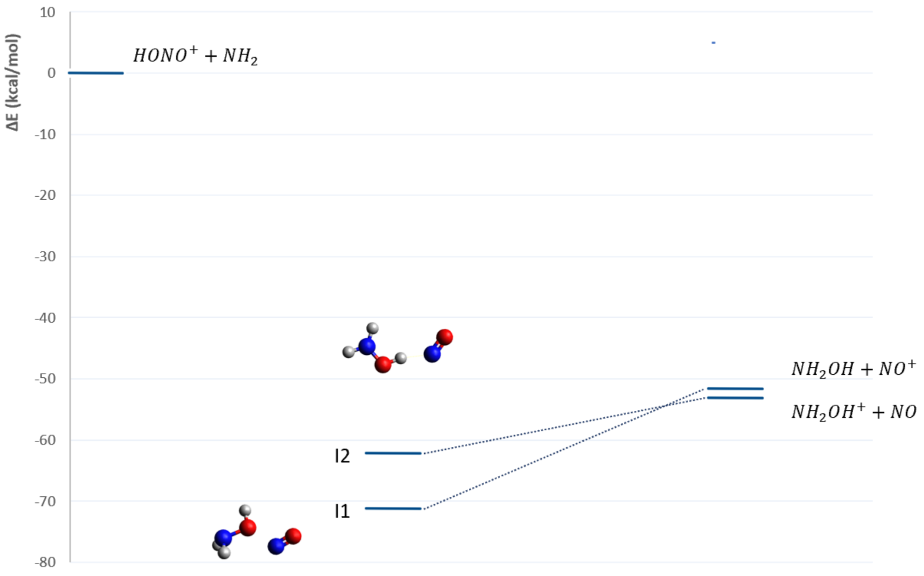

Figure S8: Thermodynamics for reaction **18**. The data are computed for a global singlet state. There is a possible path that passes through an N-O bonded complex and proceeds through the breaking of the ON bond (lowest energy path). A second path passes instead through a complex stabilized by a hydrogen bond (highest energy path). By population and orbital analysis we can speculate that **I1** is the most likely precursor of neutral hydroxylamine. The energies have been computed at the B3LYP/def2-TZVPP//CCSD(T)/def2-QZVPP level.

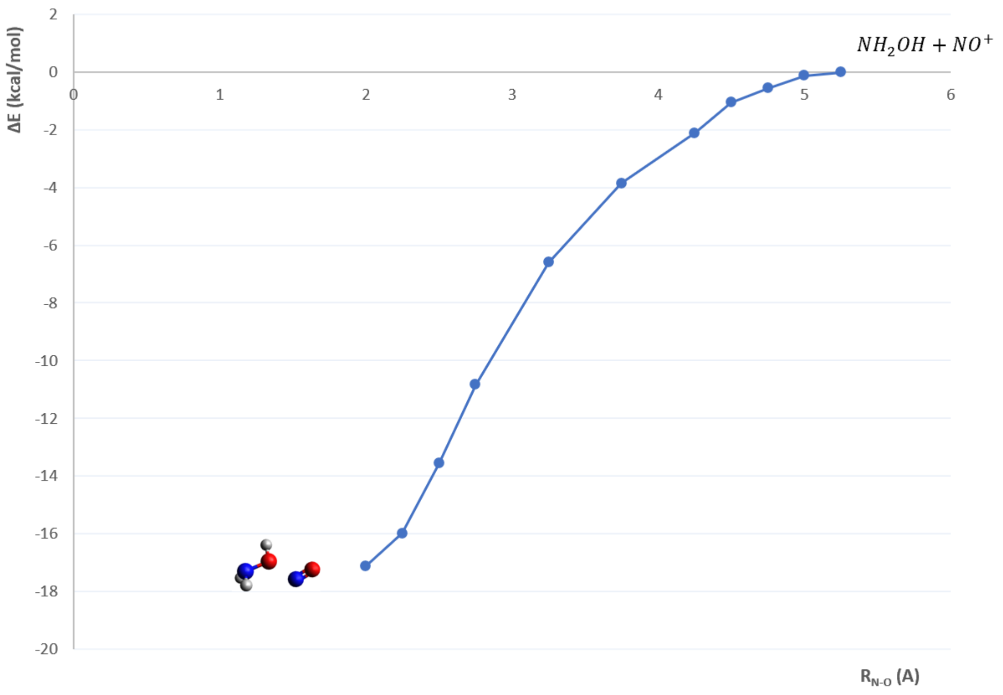

Figure S9: Electronic energy of the exit channel of reaction **18.** The geometry of the ground state complex I2 (see Figure S8) is displayed. The final products is neutral hydroxylamine. The energies have been evaluated using a CASSCF(14,12)/def2-TZVPP plus NEVPT2 correlation. The active space, albeit not including all valence orbitals, is suitable for the description of the ground state.

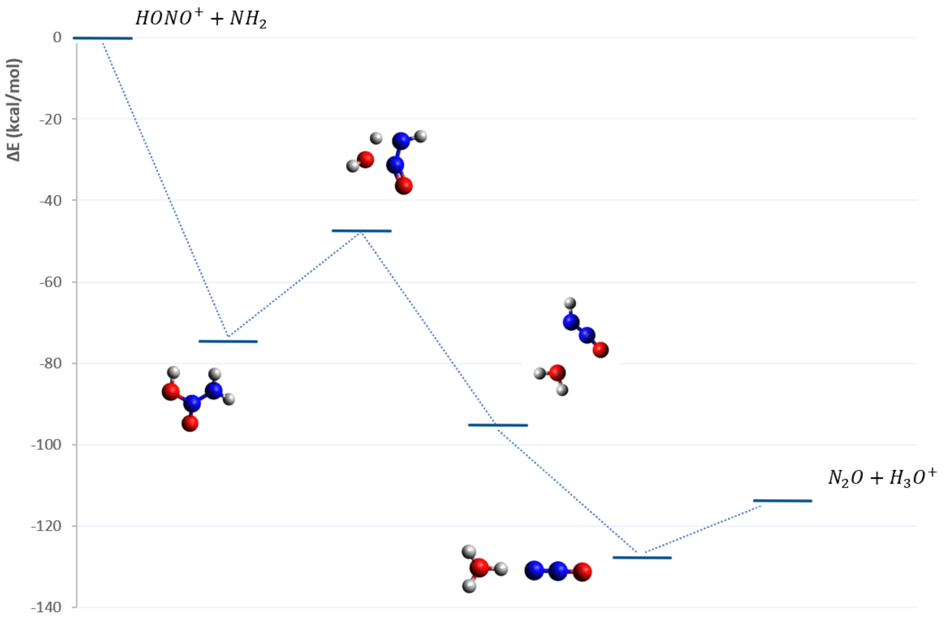

Figure S10: Alternative reactive path starting from $HONO^+ + NH_2$ on a singlet PES. The path leads to the final product through a submerged activation barrier due to the breaking of an N-O bond. The energies have been computed at the B3LYP/def2-TZVPP//CCSD(T)/def2-QZVPP level.
